# Obituary

**Published:** 1853-04

**Authors:** 


*Obituary.* — We regret to announce the death of Benjamin F. Wendel, M. D., of yellow fever, contracted in the discharge of his professional duty at Harbor Island, in the Bahamas, which occurred December 18, 1852. Dr. Wendel was one of our diligent and valued Assistant Physicians at Belleveu Hospital, in 1847 and 1848, when he nearly sacrificed his life in that service, having suffered an attack of ship fever of great severity and danger. He was a graduate, we believe, of the University of Louisville, Kentucky, and had just attained the age of twenty-six years. He was a clear headed and sound hearted man, and promised to take a high position in his profession. We loved him for his truthfulness, fidelity and honor, and sorrow most of all that we shall see his face no more on earth. Peace to his memory.—*N. Y. Med. Gazette.*

---

*Concours in France.* — It is stated that Napoleon III. has abolished the system of *concours*, and that medical professors and hospital physicians are hereafter to be appointed by himself.

